# Coulomb engineering of the bandgap and excitons in two-dimensional materials

**DOI:** 10.1038/ncomms15251

**Published:** 2017-05-04

**Authors:** Archana Raja, Andrey Chaves, Jaeeun Yu, Ghidewon Arefe, Heather M. Hill, Albert F. Rigosi, Timothy C. Berkelbach, Philipp Nagler, Christian Schüller, Tobias Korn, Colin Nuckolls, James Hone, Louis E. Brus, Tony F. Heinz, David R. Reichman, Alexey Chernikov

**Affiliations:** 1 Departments of Physics and Electrical Engineering, Columbia University, New York, New York 10027, USA; 2 Department of Chemistry, Columbia University, New York, New York 10027, USA; 3 Department of Applied Physics, Stanford University, Stanford, California 94305, USA; 4 SLAC National Accelerator Laboratory, Menlo Park, California 94025, USA; 5 Departamento de Física, Universidade Federal do Ceará, Caixa Postal 6030, Ceará, Fortaleza 60455-760, Brazil; 6 Department of Mechanical Engineering, Columbia University, New York, New York 10027, USA; 7 Department of Chemistry and James Franck Institute, University of Chicago, Chicago, Illinois 60637, USA; 8 Department of Physics, University of Regensburg, Regensburg D-93040, Germany

## Abstract

The ability to control the size of the electronic bandgap is an integral part of solid-state technology. Atomically thin two-dimensional crystals offer a new approach for tuning the energies of the electronic states based on the unusual strength of the Coulomb interaction in these materials and its environmental sensitivity. Here, we show that by engineering the surrounding dielectric environment, one can tune the electronic bandgap and the exciton binding energy in monolayers of WS_2_ and WSe_2_ by hundreds of meV. We exploit this behaviour to present an in-plane dielectric heterostructure with a spatially dependent bandgap, as an initial step towards the creation of diverse lateral junctions with nanoscale resolution.

## Abstract (web summary)

Electronic bandgap tuning in semiconductors enables key functionalities in solid-state devices. Here, the authors present a strategy to control the bandgap of atomically thin WS_2_ and WSe_2_ semiconductors via manipulation of the surrounding dielectric environment rather than by modifications of the materials themselves.

The precise and efficient manipulation of electrons in solid-state devices has driven remarkable progress across fields from information processing and communication technology to sensing and renewable energy. The ability to engineer the electronic bandgap is crucial to these applications^1^. Several methods currently exist to tune a material's bandgap by altering, for example, its chemical composition, spatial extent (quantum confinement), background doping or lattice constant via mechanical strain^2^. Such methods are typically perturbative in nature and not suitable for making arbitrarily shaped, atomically sharp variations in the bandgap. Consequently, there is a motivation to approach this important problem from a fresh perspective.

The emerging class of atomically thin two-dimensional (2D) materials derived from bulk van der Waals crystals offers an alternative route to bandgap engineering. Within the family of 2D materials, much recent research has focused on the semiconducting transition-metal dichalcogenides (TMDCs)—MX_2_ with M=Mo, W and X=S, Se, Te^3^. In the monolayer limit, these TMDCs are direct-bandgap semiconductors with the optical gap in the visible and near-infrared spectral range^4^,^5^. They combine strong inter- and intraband light-matter coupling^6^,^7^ with intriguing spin-valley physics^8^,^9^,^10^, high charge carrier mobilities^11^,^12^, ready modification of the in-plane material structure^13^,^14^,^15^,^16^ and seamless integration into a variety of van der Waals heterostructures^17^.

Importantly, the Coulomb interactions between charge carriers in atomically thin TMDCs are remarkably strong^18^,^19^,^20^,^21^. This leads to a significant renormalization of the electronic energy levels and increase in the quasiparticle bandgap. The Coulomb interactions are also reflected in the binding energies of excitons, that is, bound electron–hole pairs^2^, that are more than an order of magnitude greater in TMDC monolayers than in typical inorganic semiconductors^22^,^23^,^24^,^25^,^26^. The strength of the Coulomb interaction in these materials originates from weak dielectric screening in the 2D limit^21^,^27^,^28^. For distances exceeding few nanometres, the screening is determined by the immediate surroundings of the material, which can be vacuum or air in the ideal case of suspended samples. More generally, the interaction between charge carriers is highly sensitive to the local dielectric environment^23^,^24^,^26^,^27^,^28^,^29^,^30^,^31^,^32^, as seen in measured changes of the exciton Bohr radius^33^ and in theoretical analysis of the environmental screening^34^,^35^. Correspondingly, both the electronic bandgap and the exciton binding energy are expected to be tunable by means of a deliberate change of this environment, as illustrated in Fig. 1a, like the influence of a solvent on the properties of molecules, quantum dots, carbon nanotubes and other nanostructures suspended in solution^36^,^37^,^38^. In addition, passivated and chemically inert van der Waals surfaces allow atomically thin layers to be brought into close proximity while still retaining the intrinsic properties and functionality of the individual components^17^. These observations motivate a programme to explore the concept of Coulomb engineering of the bandgap by local changes in the dielectric environment. This strategy offers a means of locally tuning the energies of the electronic states in 2D materials, even allowing in-plane heterostructures down to nanometre length scales^34^. As a result, this approach not only effectively demonstrates the validity of fundamental physics with respect to the Coulomb interaction in atomically thin systems, but offers a viable opportunity to directly harness these many-body phenomena for future technology.

In this report, we provide direct experimental demonstration of control of the bandgap and exciton binding energy in 2D materials using Coulomb engineering through the modification of the local dielectric environment. By placing layers of graphene and hexagonal boron nitride above and below monolayers (1L) of WS_2_ and WSe_2_, we achieve tuning of the electronic quasiparticle bandgap, as well as of the exciton binding energy of the two TMDC monolayers by several 100's of meV. We note that graphene is particularly well-suited to demonstrate and explore the concept of dielectric heterostructures. It combines a high dielectric screening with the possibility of adding an arbitrary number of additional layers as thin as only 3 Å. Furthermore, the TMDC/graphene structures have been heavily studied recently in a variety of contexts with potential applications in optoelectronics and photovoltaics^39^,^40^,^41^,^42^. Screening is found to be maximized for just a few layers of graphene as the surrounding dielectric, suggesting that Coulomb-engineered bandgaps can be realized with a spatial resolution on the nanoscale. Moreover, an in-plane heterostructure with a spatially dependent electronic bandgap is shown to exhibit a potential well on the order of more than 100 meV. Our results are supported by calculations employing a quantum mechanical Wannier exciton model^21^. The dielectric screening leading to the bandgap renormalization can be treated in a semiclassical electrostatic framework that accounts for the underlying substrate and the nanostructured dielectric environment, which we computed using a recently developed quantum electrostatic heterostructure approach^30^.

## Results

### Coulomb engineering of monolayer WS_2_

**Figure 1:**
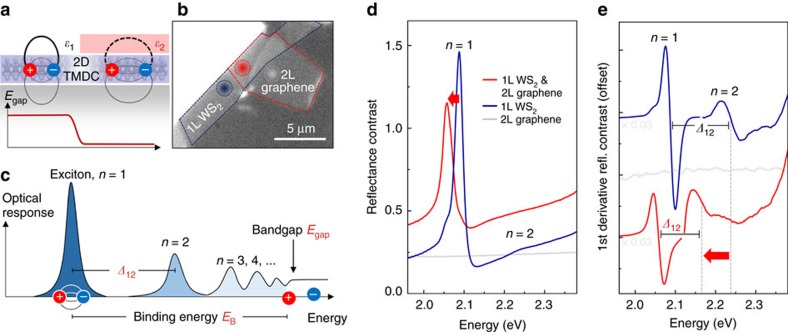
Engineering Coulomb interactions through environmental screening. (**a**) Schematic illustration of a semiconducting 2D TMDC material, partially covered with an ultra-thin dielectric layer. The strong Coulomb interaction between charged particles in low-dimensional systems affects both the exciton binding energy and the quasiparticle bandgap. The interaction can be strongly modified by modulating the environmental dielectric screening on atomic length scales. (**b**) An optical micrograph of the heterostructure under study: monolayer WS_2_ covered with a bilayer of graphene. Dotted circles indicate positions for the optical measurements. (**c**) Illustration of the optical response of an ideal 2D semiconductor, including exciton ground and excited state resonances and the onset of the (quasiparticle) bandgap. (**d**) Reflectance contrast spectra of the bare bilayer graphene, monolayer WS_2_ and the resulting WS_2_/graphene heterostructure at a temperature of 70 K. (**e**) First derivatives of the reflectance contrast spectra in **d** (after averaging over a 20 meV interval), offset for clarity. Peak positions of the exciton ground state (*n*=1) and the first excited state (*n*=2) resonances, roughly corresponding to the points of inflection, are indicated by dashed lines; Δ_12_ denotes the respective energy separations. The observed decrease of *Δ*_12_ across the in-plane boundary of the heterostructure is indicative of a reduction of the exciton binding energy and bandgap by more than 100 meV due to the presence of the adjacent graphene bilayer.

An optical micrograph of a typical sample, 1L WS_2_ partially covered with bilayer (2L) graphene, is presented in Fig. 1b. To monitor the quasiparticle bandgap of the material, we first identify the energies of the excitonic resonances in different dielectric environments using optical reflectance spectroscopy. The relationship between exciton Rydberg states and the electronic bandgap is shown in the schematic illustration of the optical response of a 2D semiconductor in Fig. 1c. The Coulomb attraction between electrons and holes leads to the emergence of bound exciton states below the quasiparticle bandgap^2^,^43^,^44^, which are labelled according to their principal quantum number *n*=1, 2, 3, …, analogous to the states of the hydrogen atom. (Throughout the rest of the manuscript we omit the term quasiparticle for clarity of presentation.) The difference between the bandgap *E*_gap_ and the exciton resonance energies defines the respective exciton binding energies. In particular, the energy *Δ*_12_ between the exciton ground state (*n*=1) and the first excited state (*n*=2) scales with the ground state exciton binding energy *E*_B_. Along with the experimentally determined transition energy *E*_1_ of the exciton ground state, we can determine the electronic bandgap via *E*_gap_=*E*_1_+*E*_B_.

Typical linear reflectance contrast spectra, Δ*R*/*R*=(*R*_sample_−*R*_substrate_)/*R*_substrate_, of the bare 2L graphene, 1L WS_2_ and the resulting heterostructure at *T*=70 K are presented in Fig. 1d. For such ultra-thin layers with moderate reflectance contrast signals on transparent substrates, the quantity Δ*R*/*R* is predominantly determined by the imaginary part of the dielectric function, which is proportional to the optical absorption^45^,^46^. In the spectral region shown, the response of 1L WS_2_ is dominated by the creation of so-called *A* excitons at the fundamental optical transition in the material, at the *K* and *K*′ points of the hexagonal Brillouin zone. In particular, the ground state (*n*=1) excitonic resonance occurs at 2.089 eV. The first excited state *n*=2 appears as a smaller spectral feature at 2.245 eV, with an energy separation between the two states of *Δ*_12_=156 meV. In addition, the first derivatives of Δ*R*/*R* are presented in Fig. 1e, where the spectral region in the range of the *n*=1 state is scaled by factor 0.03 for better comparison. Here, the energies of the peaks correspond to the points of inflection of the asymmetric derivative features, as indicated by dashed lines for the *n*=2 states. Finally, the shoulder on the low-energy side of the *n*=1 peak at 2.045 eV arises from charged excitons, indicating slight residual doping in the WS_2_ material^47^,^48^. Overall, the 1L WS_2_ response matches our previous observations on uncapped samples supported on fused silica^24^,^47^, consistent with an exciton binding energy on the order of 300 meV. For bilayer graphene, we recover the characteristic flat reflectance contrast over the relevant spectral range^49^.

In case of WS_2_ capped with graphene, the overall reflectance contrast is offset by the graphene reflectance, similar to findings in TMDC/TMDC heterostructures^50^. Most importantly, however, we observe pronounced shifts of the WS_2_ exciton resonances to lower energies, where the *n*=1 transition and the *n*=2 states are now located at 2.060 eV and 2.167 eV, respectively (Fig. 1d,e). The corresponding decrease of *Δ*_12_ from 156 to 107 meV is indicative of a strong reduction in the exciton binding energy and bandgap. In particular, the absolute shift of the *n*=2 state by almost 70 meV defines the minimum expected decrease in the bandgap. More quantitatively, by assuming a similar non-hydrogenic scaling like that in ref. 24, that is, 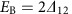, the reduction in exciton binding energy is estimated to be on the order of 100 meV, from 312 meV in bare WS_2_ to 214 meV in WS_2_ capped by 2L graphene. From *E*_gap_=*E*_1_+*E*_B_, we infer a bandgap for bare WS_2_ of 2.40 eV, reducing to 2.27 eV in the WS_2_/graphene heterostructure. We thus see a 130 meV decrease in the bandgap energy from the presence of the capping layer.

To understand these experimental findings more intuitively, we recall that although the excitons are confined to the WS_2_ layer, the electric field between the constituent electrons and holes permeates both the material and the local surroundings (Fig. 1a). In particular, the screening for larger electron–hole separations is increasingly dominated by the dielectric properties of the environment. Therefore, the strength of the Coulomb interaction is reduced by the addition of graphene layers on top of WS_2_, leading to a decrease in both the exciton binding energy and the bandgap.

### Nanoscale sensitivity of Coulomb engineering

**Figure 2:**
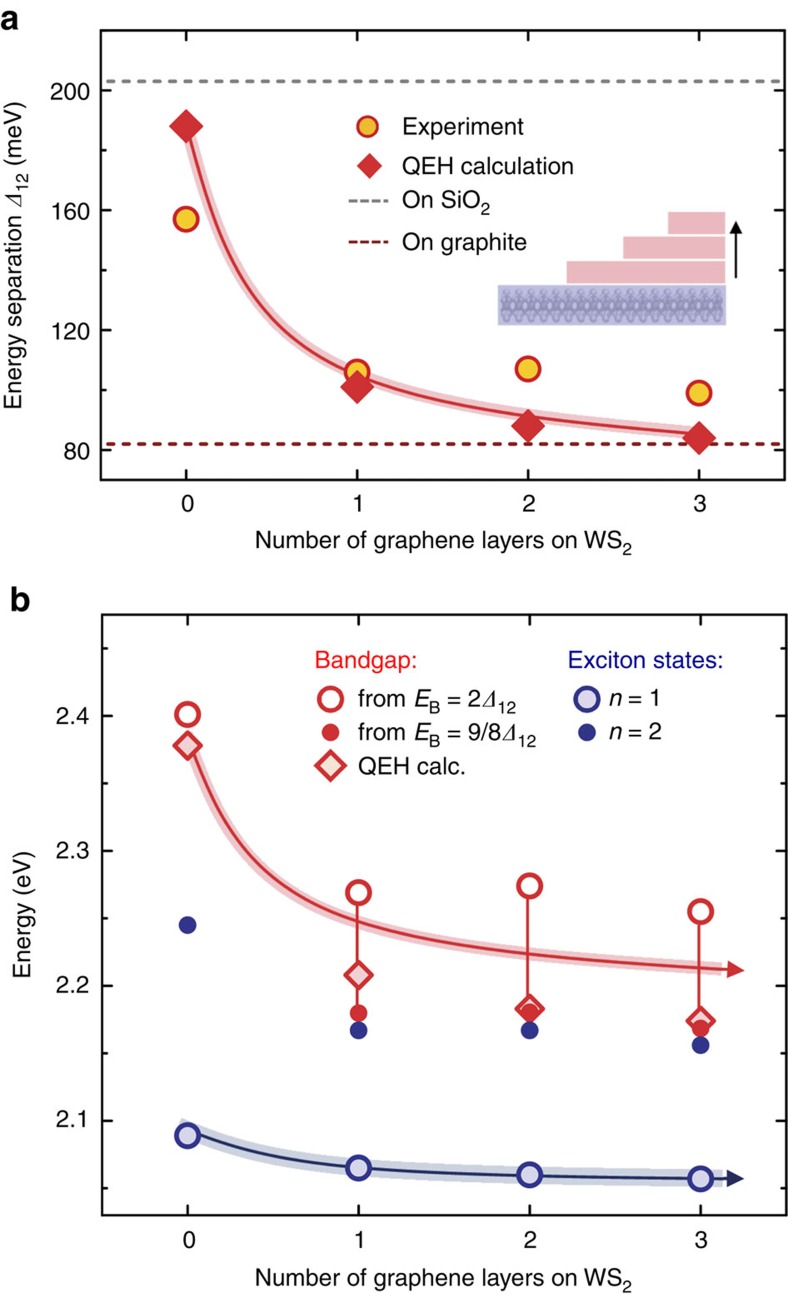
Out-of-plane spatial sensitivity of environmental screening. (**a**) Experimentally and theoretically obtained energy separation *Δ*_12_ between the *n*=1 and *n*=2 exciton states as a function of the number of layers of capping graphene. Dashed lines indicate *Δ*_12_ values from the solution of the electrostatic model for uncapped WS_2_ supported by fused silica substrate (grey) and covered with bulk graphite (red), representing two ideal limiting cases. (**b**) Absolute energies of the experimentally measured exciton ground state (*n*=1) and the first excited state (*n*=2) resonances, as well as the estimated positions of the bandgap obtained by adding the exciton binding energy to the energy of the *n*=1 state. The binding energy scales with Δ_12_, where the limiting cases are an experimentally determined non-hydrogenic scaling for WS_2_ on SiO_2_ substrate from ref. 24 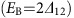 and the 2D-hydrogen model in a homogeneous dielectric 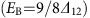. These are compared to the bandgap energies deduced from the calculated exciton binding energies using the QEH model. The solid lines are guides to the eye.

The spatial extent of the modulation is an important aspect of our approach to dielectric bandgap engineering. We have been able to probe this issue spectroscopically with sub-nanometre precision. To do so, we track the change in the WS_2_ bandgap for dielectric screening when the semiconductor is capped by 1, 2 or 3 layer graphene (Supplementary Note 3 and Supplementary Fig. 5). The extracted exciton peak separation energy *Δ*_12_ and the corresponding evolution of the bandgap are presented in Fig. 2a,b, respectively. Remarkably, we observe the strongest change already from the first graphene layer, which is followed by rapid saturation with increasing thickness within experimental uncertainty. This result strongly suggests that the change in bandgap should also occur on a similar ultra-short length scale at the in-plane boundary of the uncapped and graphene-capped WS_2_, consistent with predictions from ref. 34.

For a more precise analysis of our findings we turn to a Wannier-like exciton model^21^, where the non-local screening of the electron–hole Coulomb interaction leads to environmental sensitivity. To atomistically handle complex dielectric environments, we employ the recently introduced quantum electrostatic heterostructure (QEH) approach presented in ref. 30. Within this model, the electrostatic potential between electrons and holes confined to a 2D layer can be obtained for nearly arbitrary vertical heterostructures, taking into account the precise alignment of the individual materials and the resulting spatially dependent dielectric response. The exciton states are subsequently calculated by solving the Wannier equation in the effective mass approximation with an exciton reduced mass of 0.16 *m*_0_ as obtained from *ab initio* calculations^21^. To account for the dielectric screening from the environment, mainly through the underlying fused silica substrate and potential adsorbates such as water, we adjust the effective dielectric screening below the 2D layer, resulting in 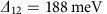 and *E*_B_=289 meV, roughly matching experimental observations. Then, additional graphene layers are added on top of the WS_2_ monolayer with all parameters being fixed. The interlayer separation between WS_2_ and graphene is set to 0.5 nm, corresponding to the average of the interlayer separations for the materials in literature^51^,^52^.

The theoretically predicted energy separation *Δ*_12_ is plotted in Fig. 2a as a function of the number of graphene layers and compared to experiment. The calculations reproduce both the abrupt change and the subsequent saturation of *Δ*_12_ with graphene thickness. Furthermore, the absolute energy values are in semi-quantitative agreement with the measurements, supporting the attribution of the measured change of *Δ*_12_ to the dielectric screening from adjacent graphene layers. The model also agrees with a classical electrostatic screening theory for the limiting cases of an uncapped WS_2_ monolayer on fused silica and for a layer fully covered with bulk graphite on top, the results of which are indicated by dashed lines in Fig. 2a (see Supplementary Note 2 for details). The calculated exciton binding energy changes from 290 meV for uncapped WS_2_ to 120 meV for the case of a trilayer graphene heterostructure. As previously discussed, the binding energies together with the absolute energies of the exciton ground state resonances can be used to infer the size of the bandgap. The evolution of the bandgap and the corresponding *n*=1 and *n*=2 exciton transition energies are presented in Fig. 2b. The binding energies obtained from the QEH model are compared with experimentally determined limits from the relation *E*_B_∝*Δ*_12_ by assuming a non-hydrogenic scaling 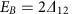 as was observed for a single WS_2_ layer on SiO_2_ (ref. 24) or conventional 2D hydrogenic scaling with 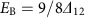 for a homogeneous dielectric. These two relations provide, respectively, boundaries for the scaling in generic heterostructures of 1L TMDCs embedded in a dielectric environment with higher dielectric screening than the SiO_2_ support and lower dielectric screening than the corresponding bulk crystals. In general, the scaling of *E*_B_ with *Δ*_12_ converges towards the 2D-hydrogen model as the screening of the surroundings approaches that of the bulk TMDC. For the case of trilayer graphene, this simple estimate implies a bandgap reduction of at least 150 meV and at most 230 meV.

### Flexibility of material systems and configurations

**Figure 3:**
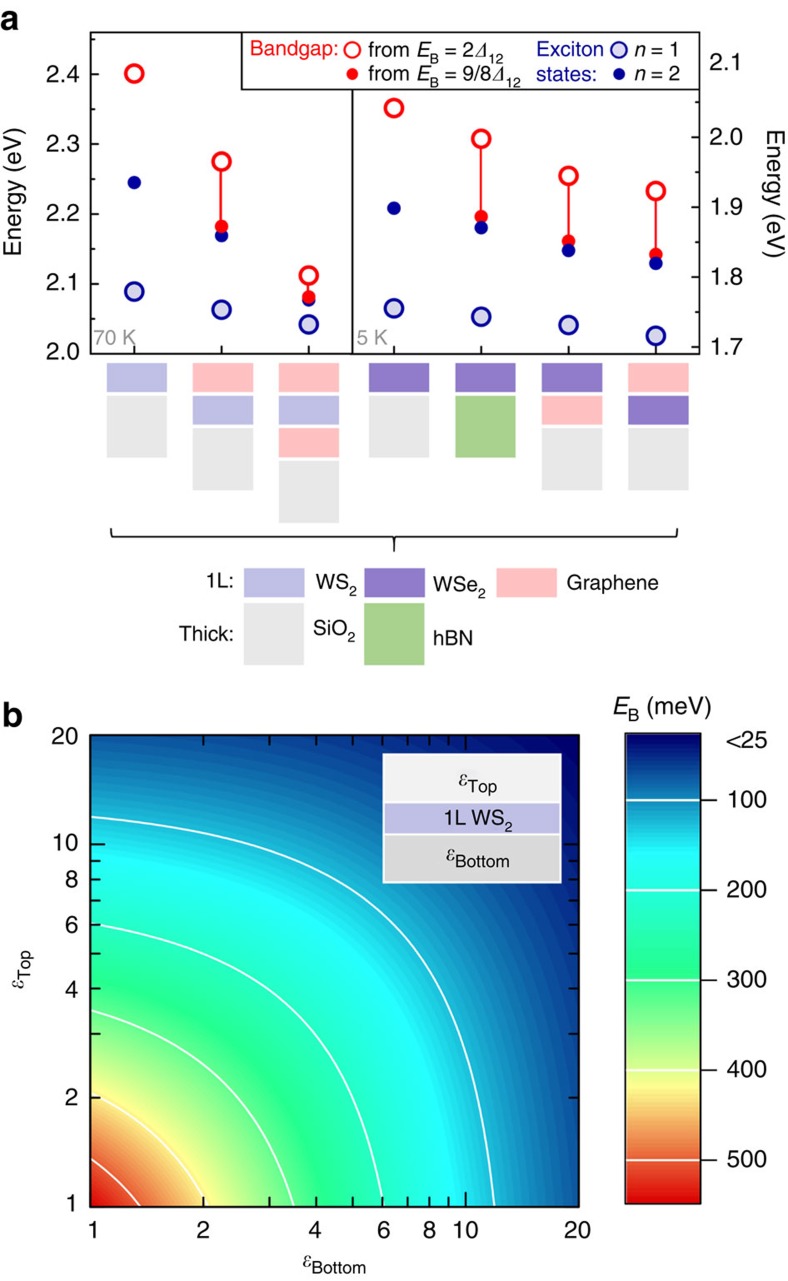
Influence of the choice and configuration of materials on the dielectric tuning of the bandgap. (**a**) Experimentally measured exciton ground state (*n*=1) and the first excited state (*n*=2) transition energies, as well as the estimated shifts of the bandgap for a variety of heterostructures. Their respective stacking configurations are indicated along the horizontal axis. The bandgap is obtained by adding the exciton binding energy to the measured transition energy of the *n*=1 state. To estimate the binding energy from the energy separation of the exciton states *Δ*_12_, we considered the limiting cases of a non-hydrogenic scaling from ref. 24 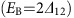 and the 2D-hydrogen model 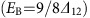. (**b**) An overview of predicted changes in the exciton binding energy in 1L WS_2_, encapsulated between two thick layers of dielectrics. The binding energy *E*_B_ is calculated by using the electrostatic approach in the effective mass approximation and presented in a 2D false-colour plot as a function of the top and bottom dielectric constants. The changes in the magnitude of *E*_B_ are roughly equal to the corresponding shifts of the bandgap and can reach 500 meV.

In addition to the graphene-capped WS_2_ samples, a variety of heterostructures were investigated in a similar manner. These include 1L WS_2_ encapsulated between two graphene layers, graphene-capped 1L WSe_2_, graphene-supported 1L WSe_2_ and 1L WSe_2_ on an 8 nm layer of hexagonal boron nitride (hBN). In all cases, a decrease in *Δ*_12_ separation was observed with increasing dielectric screening of the environment (see Supplementary Notes 4 and 5 including Supplementary Figs 6 and 7 as well as the Supplementary Table 1 for individual reflectance spectra and additional sample details). A summary of the results is presented in Fig. 3a, including experimentally obtained *n*=1 and *n*=2 transition energies, as well as the corresponding shifts of the bandgap, estimated as above (see also Supplementary Fig. 8 for the shift of the B exciton states in WSe_2_). The bandgap of WSe_2_ can be thus tuned by more than 100 meV and the largest shift of almost 300 meV is observed for graphene-encapsulated WS_2_, the structure with the highest dielectric screening. For comparison, the influence of an arbitrary dielectric environment is presented in Fig. 3b, which shows the calculated exciton binding energy of 1L WS_2_ encapsulated between two thick layers of varying dielectric constants. As we have shown, the change in the bandgap is roughly the same as the change in the binding energy and thus can be as high as 500 meV (corresponding to the intrinsic value of the exciton binding energy for a sample suspended in vacuum).

### In-plane dielectric heterostructure

**Figure 4:**
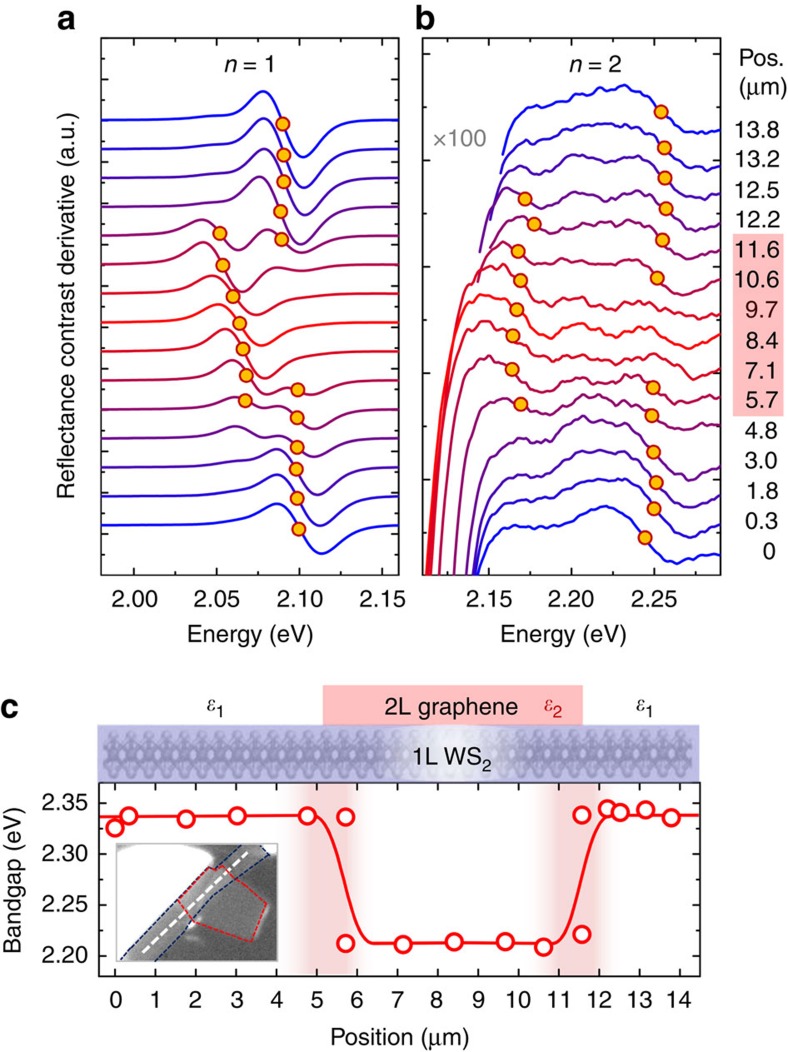
In-plane heterostructure via Coulomb engineering of monolayer WS_2_. (**a**) First-order derivatives of the reflectance contrast of a 1L WS_2_ sample for varying spatial positions across the lateral 1L WS_2_/2L graphene boundary. The data are shown in the spectral range of the exciton ground state (*n*=1) resonance and vertically offset for clarity. (**b**) For the spectral range of the excited state (*n*=2) of the exciton with the vertical axis scaled by factor of 100 for direct comparison. Full circles in **a**,**b** indicate peak energies of the resonances, corresponding to the points of inflection of the derivatives. (**c**) Spatially dependent bandgap energy extracted from the exciton peak positions along the profile of the lateral WS_2_/graphene heterostructure, as illustrated in the schematic representation and marked by the dashed line in the optical micrograph. The shaded areas indicate the diffraction limit corresponding to the spatial resolution of our measurement and the solid line is a guide to the eye.

Finally, we demonstrate an in-plane 2D semiconductor heterostructure with a spatially dependent bandgap profile by constructing a spatially varying dielectric environment surrounding the semiconductor. We scan across the structure (cf. Fig. 1b) through regions of bare WS_2_ and WS_2_ covered by a bilayer of graphene. The corresponding path is illustrated schematically in Fig. 4c and in the inset. First-order derivatives of the reflectance contrast spectra are presented in Fig. 4a,b in the spectral range of the WS_2_ exciton *n*=1 and *n*=2 resonances, respectively. Each spectral trace corresponds to a different spatial position *x* on the sample; the bilayer graphene flake covers the WS_2_ monolayer between 5 and 12 μm on the *x* axis. Like the data shown in Fig. 1d,e, both the ground and excited state resonances of the WS_2_ excitons shift to lower energies in the presence of graphene. The peak energies are extracted from the points of inflection of the derivative, indicated by circles in Fig. 4a,b. The appearance of multiple transitions in the same spectrum reflects the limited spatial resolution (1 μm) and a small amount of the WS_2_ monolayer not being in close contact with graphene (see Supplementary Note 1 and Supplementary Fig. 2 for details).

The spatial dependence is presented in Fig. 4c along the path marked in the optical micrograph (inset), which includes two WS_2_/graphene in-plane junctions. As previously discussed, the induced energy shifts result in an overall decrease of the relative energy separation *Δ*_12_ from about 160 meV, down to 105 meV. Here, the binding energy is extracted by multiplying Δ_12_ with the scaling factor deduced from the QEH calculations presented in Fig. 2 (1.54 and 1.40 for the bare and 2L graphene-covered sample, respectively) to obtain the bandgap at each point. The resulting bandgap profile is representative of a potential well (graphene-covered area) surrounded by two adjacent barriers at higher energies (bare sample). Model self-energy calculations on monolayer TMDCs in structured dielectric environments^34^ suggest that the interface between the uncapped and capped regions should yield an in-plane type-II heterostructure. In particular, the areas capped by graphene are expected to have a higher local valence band that acts as a potential well for holes. The dielectric effect on the conduction band is predicted to be weaker, with a slightly higher energy for the capped regions leading to a small barrier for electron flow from the bare to capped regions. Since the overall energy shifts of the bandgap are larger than thermal energy at room temperature, our results render the observed phenomenon technologically promising for applications under ambient or even high-temperature conditions.

## Discussion

We have demonstrated a new approach to the engineering of electronic properties through local dielectric screening of the Coulomb interaction in 2D heterostructures. We have shown tuning of the bandgap and exciton binding energy in monolayers of WS_2_ and WSe_2_ for a variety of combinations with graphene and hBN layers. The overall shift of the bandgap ranged from 100 to 300 meV, with an estimated theoretical limit of about 500 meV. In addition, the saturation of the screening effect with the thickness of the dielectric layer is found both in theory and experiment to occur on a nanometre length scale. We have demonstrated the flexibility of the technique by examining a variety of material combinations including WS_2_, WSe_2_, graphene and hBN in several distinct configurations, with top and bottom alignment as well as in a sandwich-type structure. We emphasize that the screening effect is not restricted to any particular choice of a capping material. Finally, we demonstrated Coulomb engineering of a prototypical in-plane dielectric heterostructure, illustrating the feasibility of our approach. As a consequence, we expect that patterning of dielectric layers on top of these ultra-thin semiconductors or placing the latter on a prefabricated substrate will allow us to explore a variety of novel devices in the 2D plane. In addition to the impact for more conventional optoelectronic devices—such as transistors, light emitters and detectors— one can envision custom-made superstructures for 2D layers that permit integration with photonic cavities, plasmonic nanomaterials and quantum emitters for the creation of new hybrid technologies. The considerable strength of the Coulomb forces in atomically thin materials is thus not only of fundamental importance, but also offers a strategy towards deterministic engineering of bandgaps in the 2D plane.

## Methods

### Sample preparation

Monolayers of WS_2_ and WSe_2_, mono- and few-layer graphene, and hBN samples were produced by mechanical exfoliation of bulk crystals (2Dsemiconductors and HQgraphene). The thickness of the layers was confirmed by optical contrast spectroscopy. The heterostructures were fabricated using well-established polymer-stamp transfer techniques described in refs 50, 53 for the WS_2_ based samples and ref. 54 for the WSe_2_ samples (see Supplementary Note 2 and Supplementary Fig. 1 for additional details).

### Optical spectroscopy

To study the exciton states, we performed optical reflectance measurements using a tungsten-halogen white-light source. The light was focused to a 1–2 μm spot on the sample for the measurements on WS_2_, and to a 5–10 μm spot for the measurements on WSe_2_ due to larger sample sizes. The samples were kept in an optical cryostat at temperatures around 70 K and 4 K for the WS_2_ and WSe_2_ samples, respectively. The reflected light was spectrally resolved in a grating spectrometer and subsequently detected by a CCD (see Supplementary Notes 1 and 3 and Supplementary Fig. 5 for additional details of the experimental measurements and analysis procedures).

### Theoretical methods

Exciton binding energies were calculated within the Wannier–Mott model, with an exciton reduced mass obtained from density functional theory (DFT) calculations^21^. The electron–hole-screened Coulomb interaction was obtained from the quantum electrostatic heterostructure approach^30^ (Supplementary Note 2 and Supplementary Figs 3 and 4 for additional details).

### Data availability

The datasets generated during and/or analysed during the current study are available from the corresponding author on reasonable request.

## Additional information

**How to cite this article:** Raja, A. *et al*. Coulomb engineering of the bandgap and excitons in two-dimensional materials. *Nat. Commun.* **8,** 15251 doi: 10.1038/ncomms15251 (2017).

**Publisher's note**: Springer Nature remains neutral with regard to jurisdictional claims in published maps and institutional affiliations.

## Supplementary Material

Supplementary Information Supplementary Figures, Supplementary Table, Supplementary Notes and Supplementary References
