# Supplementary material for: Coulomb engineering of the bandgap and excitons in two-dimensional materials
Source: Nat Commun. 2017 May 4;8:15251. doi: 10.1038/ncomms15251 (PMC5418602; doi:10.1038/ncomms15251)
Supplement: Supplementary Information — Supplementary Figures, Supplementary Table, Supplementary Notes and Supplementary References [file ncomms15251-s1.pdf]

Supplementary Note 1. EXPERIMENTAL METHODS

A. Sample fabrication

To prepare graphene-capped WS_2 we use a polymer based transfer technique.[1] Monolayer WS_2 was exfoliated on quartz substrates, while the graphene flakes were exfoliated directly on poly-dimethylsiloxane (PDMS) supported polymer substrates. Due to its low glass transition temperature of around 40°C, polypropylene carbonate (PPC) was the polymer of choice. The transfer procedure is described in Supplementary Figure 1. The flake in red represents the graphene layer(s) and the green flake is monolayer WS_2 on quartz. The quartz substrate is initially heated to 40°C and the flakes are continuously aligned under a microscope as the graphene approaches the WS_2. To create the lateral heterostructure we align the flakes so that some WS_2 remains uncapped. After making contact, we heat the stack to a temperature exceeding 80°C, allowing the PPC to separate from the PDMS and the graphene to be deposited on top of the WS_2. The graphene/WS_2 stack on quartz is then cooled to room temperature and soaked overnight in chloroform to dissolve the PPC. The heterostructures with WSe_2 (HQ Graphene) and hBN (2D Semiconductors) components were fabricated using an all-dry transfer technique.[2] The bulk crystals were first mechanically exfoliated onto a PDMS stamp and subsequently transferred onto an SiO_2/Si substrate using a mechanical translation stage.

B. AFM characterization of heterostructures

An atomic force micrograph of a representative heterostructure is shown in Supplementary Figure 2. The AFM image was obtained using a Bruker Dimension Fast Scan system in the non-contact Scan-Asyst mode. Region **a**, to the left of the white dashed-line, shows the topography for bare monolayer WS_2 on quartz. The WS_2 to the right of the dashed line is covered by trilayer graphene. A line scan along the white arrow at the lateral heterojunction is shown in the inset. The increase in height at the interface corresponds roughly to the thickness of trilayer graphene. The van der Waals interaction between graphene and WS_2 leads to significant areas of contact in the heterostructure, along with wrinkles and bubbles where surface contaminants accumulate. The latter regions cover roughly 10 % of the area and are discernible as high topography features in AFM images. In contrast, the bare WS_2 is smoother, as expected for an as-exfoliated sample.

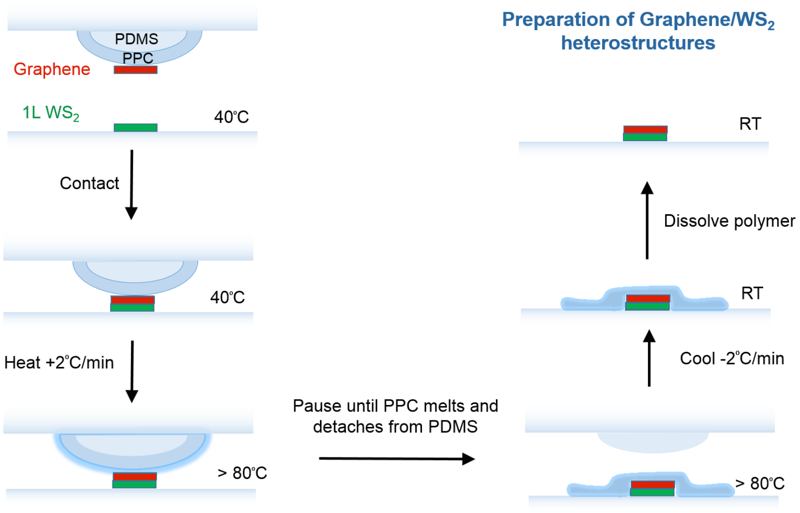

Supplementary Figure 1: **Sample fabrication.** Illustration of the transfer procedure for preparing graphene-capped WS_2.

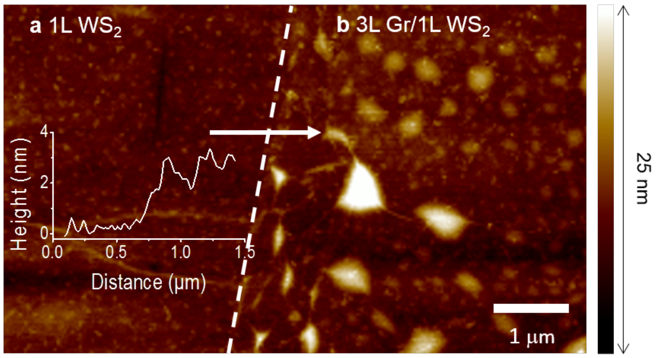

Supplementary Figure 2: **AFM micrograph** of a lateral heterojunction of **a** bare WS_2 and **b** trilayer graphene covered WS_2. The inset shows the onset of the graphene-covered region in the direction of the white arrow from region ‘a’ to ‘b’.

C. Optical reflectance measurements

For the reflectance contrast measurements of the WS_2 based samples, broadband radiation from a tungsten quartz halogen source was focused on the sample through a 40x objective, yielding a spot of about 1–2 μm in diameter. The reflected light was dispersed through a grating spectrometer and collected with a Peltier-cooled EMCCD. The measurements were performed at a temperature of about 70 K in a liquid nitrogen cooled optical cryostat. For each measurement, two reflectance contrast spectra of the sample were acquired at center wavelengths of the spectrometer of 550 nm

and 600 nm, with a spectral width of the detection window of about 150 nm. This allowed the WS_2 ground state exciton and the excited states to be sampled at two different grating positions. Any fluctuations in the overlapping spectral region arising from variation in the pixel sensitivity of the EMCCD were thus excluded in the analysis. The integration time for a single measurement (0.5s) was chosen so that the detector approached saturation. The measurements were repeated 400 times at each grating position, with a resulting averaged signal to noise ratio on the order of 10^3. For the analysis of the excited states, the raw spectra were smoothed using a moving-average over 20 pixels (roughly corresponding to a 20 meV energy interval) before and after taking a derivative. To account for any changes in the line-shape introduced by smoothing, the same procedure was applied to all curves simulated using a multi-Lorentzian model for the dielectric function and application of transfer-matrix method to calculate the resulting optical response. In case of the WSe_2 based samples, we followed a similar procedure as described above, but with a few differences. The sample was maintained at around 5 K in a liquid helium cooled cryostat, and light for the reflection contrast measurements was focused to a spot size of around 5–10 μm due to availability of larger samples. The spectra were acquired at three central energies (1.8 eV, 2.0 eV, 2.3 eV) with an integration time of 0.15 s, repeated 200 times at each grating position.

Supplementary Note 2. EXCITON BINDING ENERGY CALCULATIONS

We calculate, within the effective mass approximation, the excitonic states of monolayer WS_2 deposited on an SiO_2 substrate and covered by layers of graphene. The Wannier-Mott equation for excitons is given by

$$\left[\frac{\hbar^2}{2\mu}\nabla^2 + V(r)\right]\psi_n(r) = E_n\psi_n(r), \quad (1)$$

where r is the two-dimensional relative coordinate between electron and hole, $V(r)$ is the screened electron-hole interaction potential, $\psi_n(r)$ is the exciton envelope function, and E_n is the exciton binding energy relative to a free electorn and hole..

The reduced effective mass of the electron-hole pair in WS_2 is taken to be $\mu = 0.16m_0$, as obtained by *ab initio* calculations.[6] The theoretical description of the excitonic properties of the system requires a proper understanding of the electron-hole interaction potential and of the effect of dielectric screening by the WS_2 layer and its surrounding environment. Results for the excitonic states presented in the main manuscript are obtained with an interaction potential calculated by the Quantum Electrostatic Heterostructure model (QEH),[7] which uses in-plane averaged density re-

sponse functions $\chi_i(k_\parallel, \omega)$ (obtained separately from *ab initio* calculations for each of the materials composing a van der Waals stack of layers) as building blocks for the overall dielectric function of this stack. The inverse dielectric matrix is obtained as

$$\epsilon_{ia,jb}(k_\parallel, \omega)^{-1} = \delta_{ia,jb} + \sum_{kc} \bar{V}_{ia,kc}(k_\parallel)\chi_{kc,jb}(k_\parallel, \omega), \tag{2}$$

where indices i, j, k label the layers and $a, b, c = 0, 1$ correspond to monopole (0) and dipole (1) components. The density response matrix $\chi_{ia,jb}(k_\parallel, \omega)$ represents the monopole (dipole) density induced in the ith layer by a constant (linear) potential applied in the jth layer, as calculated by a Dyson-like equation.[7] The Coulomb matrix is obtained from the induced charge density $\rho_{ia}(z, k_\parallel)$ and the associated potential $\Phi_{kc}(z, k_\parallel)$, averaged over the thickness of the slab,

$$\bar{V}_{ia,kc}(k_\parallel) = \int \rho_{ia}(z, k_\parallel)\Phi_{kc}(z, k_\parallel)dz. \tag{3}$$

First-principles response functions used in our calculations were taken from the Computational Materials Repository.[8]

Once the dielectric matrix $\epsilon_{ia,jb}(k_\parallel, \omega)$ is obtained, the electron-hole interaction potential in reciprocal space is given by

$$V(k_\parallel) = \sum_{ia,jb,kc} \rho^e_{ia}(k_\parallel)\epsilon^{-1}_{ia,jb}(k_\parallel)\bar{V}_{jb,kc}(k_\parallel)\rho^h_{kc}(k_\parallel), \tag{4}$$

where the electron (hole) charge distribution $\rho^{e(h)}_{ia(kc)}$ is set to 1 at the WS_2 layer and to zero in the other layers, thus representing an exciton localized in WS_2. Finally, we perform an inverse Fourier transform of this potential and solve Supplementary Equation 1 in real space.

Dielectric screening due to the SiO_2 substrate cannot be taken into account exactly within the QEH model, which deals only with layered materials. Nevertheless, the substrate can be approximated by a thick stack of hexagonal boron nitride (hBN) layers, since bulk BN and SiO_2 have similar static dielectric constants ($\varepsilon \approx 4$). First-principles calculations indicate a 5.08 Å separation between WS_2 and the hBN stack, while the interlayer distance in hBN is found to be 3.22 Å.[7] The energy levels of the first four s-type excitons in WS_2 on 45 hBN layers, as obtained by the QEH model, are shown in Supplementary Figure 3, along with the exciton binding energies inferred from the experimental absorption spectrum (by subtracting the exciton peaks positions from a $E_g = 2.36$ eV quasiparticle gap) for bare WS_2 on an SiO_2 substrate in Ref. [9]. Fairly good agreement between model and experiment is observed, especially considering that the model has

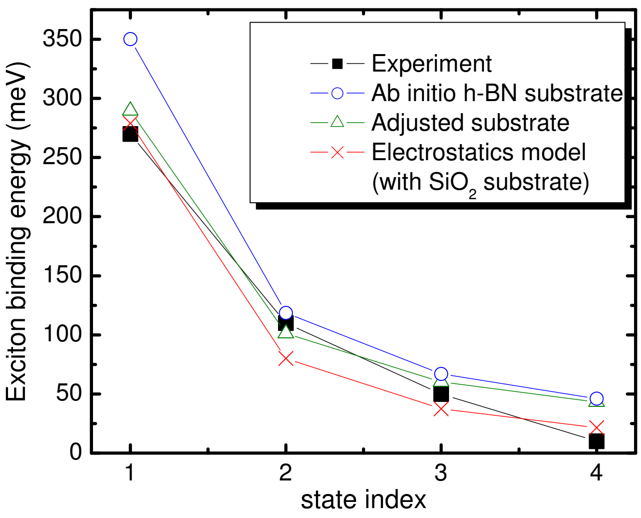

Supplementary Figure 3: **Exciton binding energies** for states n = 1 to 4 for monolayer WS_2 as obtained experimentally from Ref. 9 (by subtracting the exciton peaks positions from a $E_g = 2.36$ eV quasiparticle gap for bare WS_2 on SiO_2), along with numerical results predicted by different theoretical models described in the text.

no free parameters. The binding energy of the $n = 1$ state is overestimated by about 80 meV. To account for this, we reduce the separation between the hBN layers as well as between the hBN stack and the WS_2 layer to 2.5 Å so that the binding energy of the $n = 1$ state and the separation between $n = 1$ and $n = 2$ states are well matched to the experimental results on bare, uncapped WS_2 on SiO_2. All subsequent calculations with capping layers of graphene are performed with no additional adjustments.

We have also confirmed that our treatment of the substrate is converged with respect to the number of hBN layers. As shown in Supplementary Figure 4 (using the aforementioned 2.5 Å layer separation), the energy separation Δ_{12} and the exciton binding energy rapidly converge; beyond 40 layers, changes in these quantities are of only a few meV. Therefore, all theoretical results in the main manuscript employ a substrate consisting of 45 hBN layers.

An alternative semiclassical approach to screening can be employed, which is more naturally suited to screening via a semi-infinite insulating substrate. In this approach, one derives a screened potential as the solution to the Poisson equation for a slab of thickness d and dielectric constant ϵ in between two semi-infinite media with dielectric constants ϵ_1 and ϵ_2. For arbitrary dielectric constants, the potential in the center of the slab is given by $V(k) = -2\pi/\,[\varepsilon(k)k]$ with the dielectric

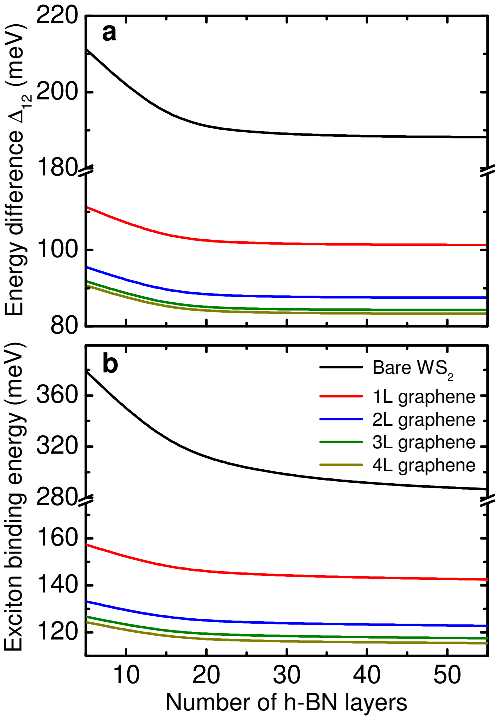

Supplementary Figure 4: **Binding energy and Δ_{12} convergence with substrate thickness. a** Calculated energy difference Δ_{12} between $n = 1$ and $n = 2$ exciton states and **b** ground state exciton binding energy of a WS_2 monolayer capped with few-layer graphene as a function of the number of hBN layers in the substrate.

function

$$\varepsilon(k) = \epsilon \frac{1 - L_1 L_2 e^{-2kd}}{(1 - L_1 e^{-kd})(1 - L_2 e^{-kd})} \tag{5}$$

where $L_n = (\epsilon_n - \epsilon)/(\epsilon_n + \epsilon)$. In the limit $kd \ll 1$ and $\epsilon \gg \epsilon_{1,2}$, the above reduces to the Keldysh form.[6,10] However, this later approximation is not necessarily accurate for a WS_2 slab (d = 5 Å, $\epsilon = 15$) capped by semi-infinite graphite ($\epsilon_1 = 10$) and supported on SiO_2 ($\epsilon_2 = 3.9$), and so the exact solution of the Poisson equation is preferred and used here.

Despite the simplicity of this model, the excitonic states resulting from this electron-hole interaction potential are found to be in satisfactory agreement with the experimental spectrum, as shown in Fig. 2a of the main text and Supplementary Figure 3. In particular, we find that the uncapped WS_2 on SiO_2 has $\Delta_{12} \approx 200$ meV, whereas WS_2 fully covered by semi-infinite graphite has $\Delta_{12} \approx 80$ meV; both values are in reasonable agreement with the experimental results for no

and many layers of graphene, respectively.

Supplementary Note 3. ANALYSIS OF THE OPTICAL RESPONSE OF BARE AND GRAPHENE-CAPPED WS_2

In this section we discuss the analysis of the exciton states in bare and graphene-capped WS_2 samples. To analyze the excited states of the excitons in more detail, low temperature spectra acquired around 70 K were processed as described in the preceding section. Supplementary Figure 5 shows the derivative spectra in the spectral region of the first-excited states as solid lines. The energies are plotted with respect to the ground state. To accurately identify the position of the excited states, we simulated the reflection-contrast spectra of the samples. The propagation of light through the multi-layered stack of graphene, WS_2, and quartz was calculated using a standard transfer-matrix method implemented in an open-source software.[3] The primary inputs for the calculation were the thicknesses of the layers and the dielectric functions. The frequency dependent dielectric functions of graphene and quartz were sourced from literature.[4,5]

The dielectric function of WS_2 was parametrized using multiple Lorentzians for the ground and first two excited excitonic states. The peak parameters of the Lorentzians were adjusted so that the derivatives of the simulated spectra match those in the experiment. The simulated derivatives are plotted as dotted lines in Supplementary Figure 5; the circles mark the central energies of the first excited state ($n = 2$). Smaller features on the higher energy side, with linewidths broader than that of the $n = 2$ states are attributed to contributions from higher excited states and onset of the band-gap continuum. Features with linewidths similar to the $n = 1$ state are assigned to the small regions with greater graphene-WS_2 interlayer distance in the AFM micrograph in Supplementary Figure 2.

Supplementary Note 4. MONOLAYER WS_2 ENCAPSULATED BY MONOLAYER GRAPHENE

In this section we describe the effect of encapsulating WS_2 with monolayers of graphene. The experimental observables are the ground and first excited states of exciton. The energy difference between them, Δ_{12}, is proportional to the exciton binding energy. The quasiparticle bandgap corresponds to the sum of the energy of the exciton ground state and its binding energy, E_B. In this analysis, we extract E_B for the limiting cases of the 2D hydrogen model ($E_B = \frac{9}{8}\Delta_{12}$) and that of

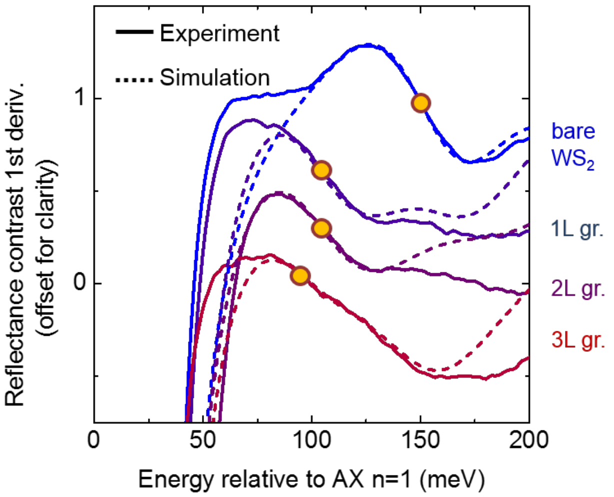

Supplementary Figure 5: **First-order derivatives of the reflectance contrast of 1L WS_2** samples capped with 0–3 layers of graphene in the spectral range of the n = 2 state of the exciton. The spectra are plotted as a function of the relative energy with respect to the n = 1 ground state resonance together with the results of the multi-Lorentzian simulation of the dielectric function. Both experimentally obtained and simulated curves are smoothed over the range of 20 meV before and after taking the derivative. The respective central energies of the n = 2 resonance are indicated by circles.

non-local screening applicable for the monolayer sample on fused silica substrate (approximated as $E_B = 2\Delta_{12}$). In the main text we report the change in Δ_{12}, binding energy and the quasiparticle gap as a function of the number of layers of graphene capping WS_2. The decrease in the binding energy and bandgap is greatest for the transition from uncapped WS_2 to one that is capped by a single layer of graphene. The expectation of an even greater change for the encapsulated sample is confirmed in the experiment described below.

An optical micrograph of the heterostructure under study is shown in Supplementary Figure 6a, and the sample geometry is described schematically in Supplementary Figure 6b. The sample was prepared by picking up a graphene-capped sample using PPC coated PDMS and transferring it on top of another flake of monolayer graphene in a manner similar to Supplementary Figure 1. The experimentally obtained reflectance contrast spectrum is plotted in red in Supplementary Figure 6c. Interference effects due to the 300 nm SiO_2/Si substrate lead to a different line-shape compared to the capped samples on fused silica that have been previously discussed. The intrinsic excitonic and

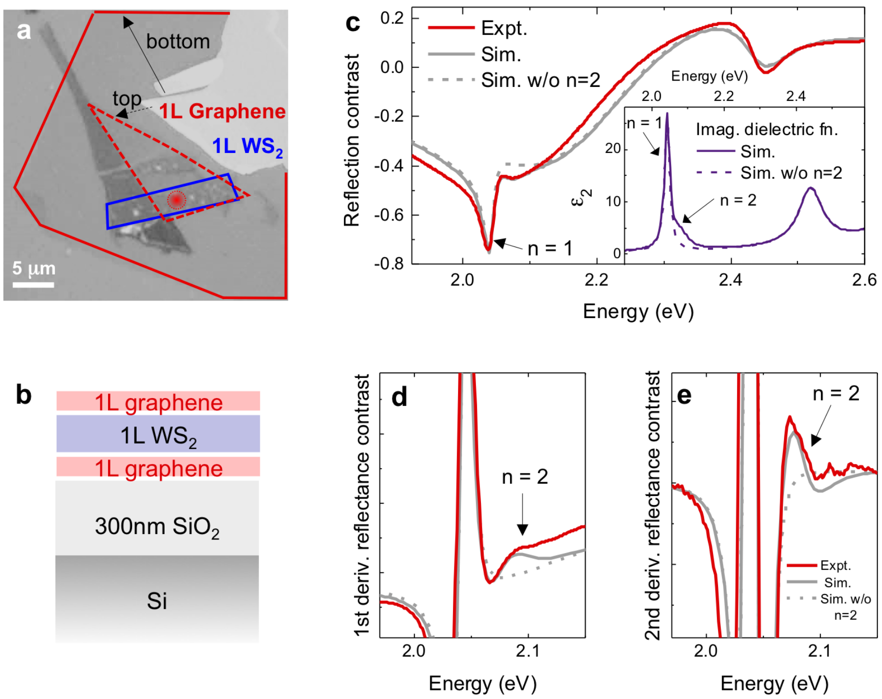

Supplementary Figure 6: **Exciton states in graphene encapsulated WS_2. a** Micrograph of monolayer WS_2 encapsulated by monolayers of graphene and supported on an SiO_2/Si substrate. **b** Schematic representation of the sample geometry. **c** Reflectance contrast spectra for WS_2 sandwiched between 1L graphene, measured in the region marked by the dotted circle in **a**. The imaginary part of the dielectric function used to to simulate the grey curves is shown in the inset, with and without and the n=2 feature. The first and second derivatives of the reflectance contrast are plotted in **d** and **e**, respectively.

electronic properties should be similar to the samples on fused silica due to the nearly identical dielectric response of thermally grown SiO_2 and the large thickness of the oxide compared to the exciton Bohr radius. The A exciton resonance is located at 2.042 eV, and simulations of the optical response were performed along the lines of the procedure described in the previous section. The solid grey line includes an extra peak on the higher energy flank of the main resonance, and for comparison we also plot a simulation in dotted grey that omits this extra feature. The peak can be visualized as a high energy shoulder of the A exciton response in the imaginary dielectric function (Supplementary Figure 6). The first and second derivatives are shown in Supplementary Figure 6d and e, respectively. The presence of the higher energy feature is most evident in the second derivative, where the the inflection at around 2.077 eV cannot be reproduced without it. The linewidth

Energy (eV) \ Sample config.	1L WS_2 / SiO_2	1L graphene / 1L WS_2 / SiO_2	1L graphene / 1L WS_2 / 1L graphene / SiO_2
Δ_{12} (expt.)	0.156	0.106	0.035
$E_{n=1}$ (expt.)	2.089	2.063	2.042
$E_B = \frac{9}{8}\Delta_{12}$	--	0.119	0.039
Bandgap (2D hyd.)	--	**2.182**	**2.081**
$E_B = 2\Delta_{12}$	0.312	0.210	0.070
Bandgap	**2.401**	**2.275**	**2.112**

Supplementary Table 1: **Excitonic and electronic properties of monolayer WS_2 for different capping geometries.** Experimentally measured Δ_{12} and $n = 1$ state energies for uncapped, graphene-capped and graphene-encapsulated 1L WS_2. All samples are supported on SiO_2 (fused silica or thermally grown-oxide). Binding energies (E_B) extracted from 2D hydrogenic and non-local models are used to calculate the lower and upper limits of the quasiparticle bandgap in conjunction with the experimentally reported $n = 1$ state energies E_1, where the quasiparticle bandgap $= E_B + E_1$

.

of the extra peak is larger than that of the main resonance and the oscillator strength is weaker by an order of magnitude, which leads us to assign it to the first excited state of the exciton.

The WS_2 used in the graphene encapsulated heterostructure was also studied in its bare and graphene-capped forms, both of which were supported on fused silica. The experimental results are summarized in Supplementary Table 1, along with and lower bounds for the exciton binding energy from the non-locally screened ($2\Delta_{12}$) and 2D hydrogenic models ($\frac{9}{8}\Delta_{12}$). The 2D hydrogen model is inapplicable in the case of the bare monolayer, as discussed in previous reports.[9,11] The quasiparticle bandgap for the encapsulated case is then expected to lie between 2.081 and 2.112 eV, corresponding to a decrease of around 300 meV compared to the case of bare monolayer WS_2.

Supplementary Note 5. EFFECT OF THE DIELECTRIC ENVIRONMENT ON 1L WSe_2

Monolayer WSe_2 is another 2D semiconductor similar to WS_2. In this section we discuss the Coulomb engineering of its bandgap by placement of neighboring layers of graphene and hexagonal boron nitride (hBN). We study three different sample geometries supported on 300 nm SiO_2/Si

substrate: 1L WSe_2 capped by 1L graphene, 1L WSe_2 on top of 1L graphene and 1L WSe_2 on thick hBN. These different structures are schematically shown in Supplementary Figure 7c, d and e, respectively. The hetrostructures were prepared by an all-dry polymer stamp transfer process (see Supplementary Note 1 A) and studied at around 5 K in an optical cryostat. Micrographs of 1L graphene capped WSe_2 and of WSe_2 on 8 nm thick hBN are shown in Supplementary Figure 7a and b. The first derivatives of the optical response of bare 1L WSe_2 monolayers are plotted in violet in Supplementary Figure 7f through h. The magnitude of the n = 1 state of the A exciton at around 1.72 eV is reduced by a factor of 2 in the presentation so that the higher exciton states can be more clearly visualized on the same scale. The separation between the ground and first excited state of the exciton (Δ_{12}) for the bare monolayer supported on SiO_2 is around 142 meV, matching the results of He et al.[11] within experimental error. This leads to a binding energy on the order of 300 meV and a corresponding electronic bandgap of around 2.04 eV at 5 K. The effect of graphene

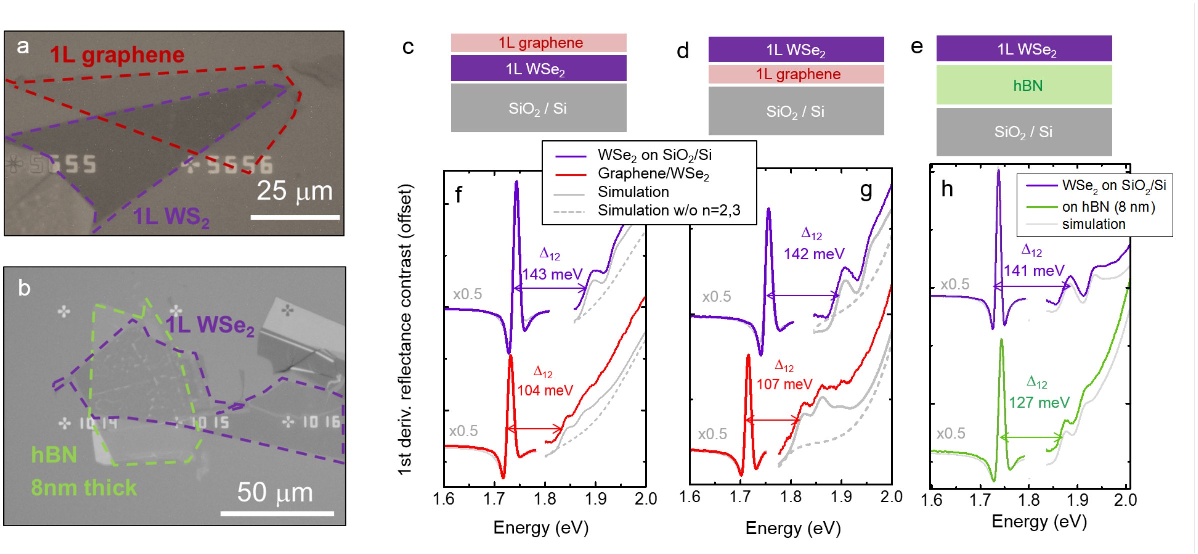

Supplementary Figure 7: **Exciton states of monolayer WSe_2 in different dielectric environments.** **a** Micrograph of monolayer WSe_2 capped by monolayer graphene, supported on an SiO_2/Si substrate. **b** Micrograph of monolayer WSe_2 placed upon 8 nm thick hBN, supported on an SiO_2/Si substrate. Schematic representations of the sample geometries, along with that of 1L WSe_2 on 1L graphene, are shown in **c** through **e**. Reflectance contrast spectra of 1L WSe_2 **f** capped with 1L graphene, **g** placed upon 1L graphene and **h** on top of 8 nm hBN. The exciton states are included in the simulation results denoted by the solid grey lines; for comparison the dotted grey show the simulations without the n = 2 and 3 states.

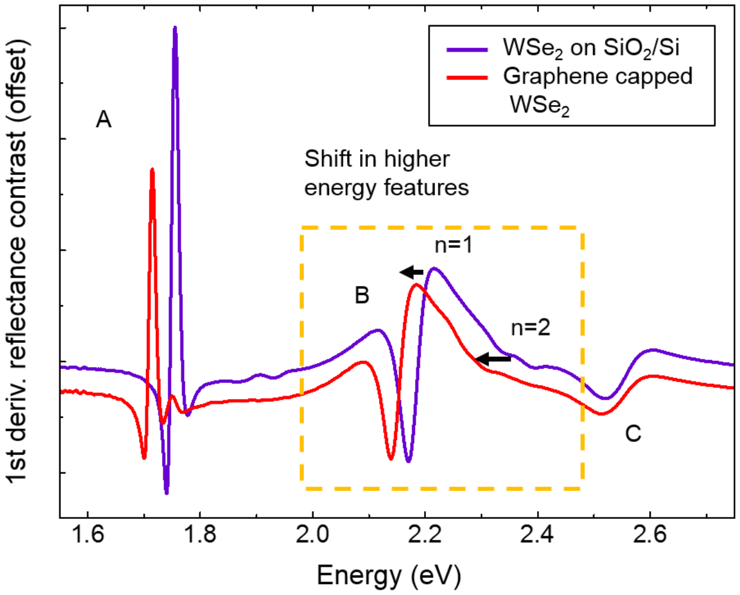

Supplementary Figure 8: **Shift in B exciton states.** The first derivative of the reflectance contrast of bare and graphene-capped WSe_2 in the spectral range from 1.6 to 2.7 eV. The A, B and C excitons are identified at around 1.7, 2.2 and 2.5 eV, with the yellow dotted line delineating the ground and excited states of the B exciton. The black arrows indicate the decrease in the energies of its n = 1 and 2 states when 1L WSe_2 is capped with graphene.

as a proximal screening media is similar to its effect on 1L WS_2, with Δ_{12} decreasing by around 40 meV. The corresponding decrease in the exciton binding energy would be on the order of a 100 meV, with the non-local screening model and 2D hydrogen model constituting the limiting cases, as discussed in Fig. 3 of the main text. When 1L WSe_2 is placed on hBN, Δ_{12} decreases by around 14 meV, translating to a decrease in the binding energy (and bandgap) on the order of 30 meV relative to the case on an SiO_2 substrate. This matches our expectations from electrostatic considerations, where the static dielectric function of graphene is greater than that of hBN, and hBN in turn has a slightly larger dielectric constant than SiO_2.

We have so far investigated the lowest energy optical transition, often referred to as the A exciton. Strong spin-orbit coupling in the tungsten atom breaks the degeneracy of energy bands, leading to additional transitions that can be accessed via linear spectroscopy techniques such as white-light reflectance. The transition at around 2.2 eV, arising from the split valence band at the K point, is referred to as the B exciton. Additional transitions at even higher energies are labeled as the C band.

The first derivatives of the reflectance contrast for bare and 1L graphene-capped 1L WSe_2 samples are plotted in Supplementary Figure 8 for the energy range from 1.6 to 2.7 eV, revealing the A, B and C transitions. Higher energy features of the B exciton around 2.3–2.4 eV were observed in both samples; we attribute these to the excited states of the B exciton due to their weak oscillator strength and larger linewidth. Like the excited states of the A exciton, these features also respond to changes in the dielectric environment. An approximately 40 meV decrease in the Δ_{12} is measured in case of capping 1L WSe_2 with graphene. This closely follows the change measured for the A exciton, corresponding to a roughly 100 meV reduction in the quasiparticle bandgap associated with the B exciton.

---

[1] Rigosi, A. F., Hill, H. M., Li, Y., Chernikov, A., & Heinz, T. F. Probing interlayer interactions in transition metal dichalcogenide heterostructures by optical spectroscopy: MoS_2/WS_2 and $MoSe_2/WSe_2$. *Nano Lett.* **15**, 5033–5038 (2015).

[2] Castellanos-Gomez, A. *et al.* Deterministic transfer of two-dimensional materials by all-dry viscoelastic stamping. *2D Mater.* **1**, 011002 (2014).

[3] Byrnes S., *Multilayer thin film optics calculator* (2012), URL http://sjbyrnes.com/.

[4] Weber, J. W., Calado, V. E., & van de Sanden, M. C. M. Optical constants of graphene measured by spectroscopic ellipsometry. *Appl. Phys. Lett.* **97**, 091904 (2010).

[5] Malitson, I. H. Interspecimen comparison of the refractive index of fused silica. *J. Opt. Soc. Am.* **55**, 1205–1209 (1965).

[6] Berkelbach, T. C., Hybertsen, M. S., & Reichman, D. R. Theory of neutral and charged excitons in monolayer transition metal dichalcogenides. *Phys. Rev. B* **88**, 045318 (2013).

[7] Andersen, K., Latini, S., & Thygesen, K. S. Dielectric genome of van der Waals heterostructures. *Nano Lett.* **15**, 4616–4621 (2015).

[8] Rasmussen, F. A, & Thygesen, K. S. Computational 2D materials database: electronic structure of transition-metal dichalcogenides and oxides. *J. Phys. Chem. C* **119**, 13169–13183 (2015).

[9] Chernikov, A. *et al.* Exciton binding energy and nonhydrogenic Rydberg series in monolayer WS_2. *Phys. Rev. Lett.* **113**, 076802 (2014).

[10] Keldysh, L. V. Coulomb interaction in thin semiconductor and semimetal films. *JETP Lett.* **29**, 658–661 (1979).

[11] He, K. *et al.* Tightly bound excitons in monolayer WSe_2. *Phys. Rev. Lett.* **113**, 026803 (2014).
